# The Utility of CO_2_ Laser Treatment of Pelvic Symptoms in Women with Previous Perineal Trauma during Delivery

**DOI:** 10.3390/jpm14010060

**Published:** 2023-12-30

**Authors:** Maurizio Filippini, Roberto Angioli, Daniela Luvero, Margaret Sammarini, Giovanna De Felice, Silvia Latella, Neila Maria de Góis Speck, Miriam Farinelli, Francesco Giuseppe Martire, Ferdinando Antonio Gulino, Giosuè Giordano Incognito, Stella Capriglione

**Affiliations:** 1 Department of Obstetrics and Gynecology, Hospital State of Republic of San Marino, 47893 Borgo Maggiore, San Marino; mfilippini@omniway.sm (M.F.); margaret.sammarini@gmail.com (M.S.); giodefelice@hotmail.com (G.D.F.); silvialatella@yahoo.it (S.L.); miriam.farinelli@iss.sm (M.F.); 2 Department of Obstetrics and Gynecology, Campus Bio-Medico University, 00128 Rome, Italy; r.angioli@unicampus.it (R.A.); d.luvero@unicampus.it (D.L.); 3 Gynecological Disease Prevention Nucleus (NUPREV), Department of Gynecology, Paulista Medical School (UNIFESP/EPM), Federal University of São Paulo, São Paulo 04023-062, Brazil; neia@hotmail.com; 4 Gynecological Unit, Department of Surgical Sciences, University of Rome “Tor Vergata”, 00133 Rome, Italy; francescogmartire@libero.it; 5 Unit of Gynecology and Obstetrics, Department of Human Pathology of Adults and Developmental Age, “G. Martino” University Hospital, 98122 Messina, Italy; 6 Department of General Surgery and Medical Surgical Specialties, University of Catania, 95123 Catania, Italy; giordanoincognito@gmail.com; 7 Department of Obstetrics and Gynecology, “Santa Maria alla Gruccia” Hospital, 52025 Montevarchi, Italy; scapriglione2016@gmail.com

**Keywords:** CO_2_ laser, delivery, episiotomy, perineal laceration, vulvovaginal atrophy

## Abstract

This study aimed to examine the impact of fractional CO_2_ laser treatment of pelvic symptoms in women who have undergone perineal trauma from vaginal delivery. It was a retrospective, monocentric analysis that encompassed all women assessed for pelvic discomfort or signs of vulvovaginal atrophy following vaginal delivery between 2013 and 2018. The severity of symptoms was assessed using the Visual Analogue Scale (VAS). Twenty-seven patients met the inclusion criteria and were sorted into two groups: (1) women who had undergone episiotomies during labor (*n* = 11); and (2) women who had experienced spontaneous tears during vaginal delivery (*n* = 16). For women with episiotomies, each treatment and subsequent evaluation consistently showed a significant reduction in dyspareunia intensity. A similar positive trend was observed regarding pain at the introitus (7.5 vs. 6.5 after the first treatment, *p* = 0.03; 6.5 vs. 3 after the second treatment, *p* = 0.01; 3 vs. 1 after the third treatment, *p* = 0.01). Among women experiencing spontaneous perineal tears during delivery, there was a notable decrease in dyspareunia following all treatments (8 vs. 7 after the first treatment, *p* = 0.01; 8 vs. 4 after the second treatment, *p* = 0.02; 3 vs. 1 after the third treatment, *p* = 0.03). The impact of laser treatment did not exhibit significant differences between women who underwent episiotomies and those who experienced spontaneous perineal tears. In conclusion, fractional CO_2_ laser can be regarded as a non-pharmacological option for managing pelvic floor symptoms in women who encountered perineal trauma during delivery, independently from the nature, spontaneity, or iatrogenesis of the perineal laceration.

## 1. Introduction

Symptoms affecting the pelvic region often arise after giving birth [1,2]. These issues are commonly linked to vulvovaginal atrophy (VVA), stemming from a sharp and swift decline in estrogen levels during the postpartum phase. In addition, pelvic floor disturbances may arise from perineal trauma associated with either spontaneous perineal tears or episiotomy. Furthermore, the process of postpartum healing varies among women. Some individuals may experience more significant changes to the vaginal tissues, including thinning and drying, which are characteristics of VVA. Many women report symptoms such as vaginal dryness, sensations of burning, discomfort, or itching, and pain during sexual activity [3]. It is noteworthy that the incidence of acute pain in the genital and pelvic region following childbirth can be remarkably high, with rates reaching up to 85% one day post-cesarean delivery [4] and 92% following a vaginal birth [2]. Typically, this pain subsides over the first two to three months postpartum, as the body recovers from the immediate injuries sustained during childbirth [5]. However, this symptom persists in some cases beyond this time frame, ranging from 4% to 27% at three months [6,7,8,9] and from 1% to 33% at one year postpartum [10,11,12,13,14]. VVA, also known as atrophic vaginitis, primarily affects women during and post-menopause, marked by the thinning, drying, and inflammation of the vaginal walls due to reduced estrogen levels. This hormonal decline, particularly significant during menopause, triggers various VVA symptoms. The condition is characterized by morphological and clinical changes, including alterations in the ratio of collagen type I to type III fibrils, a decrease in elastic fibers, diminished vascularization, and the thinning and flattening of the vaginal epithelium [15]. One of the hallmark symptoms is a persistent lack of moisture in the vaginal area, leading to discomfort and irritation. The thinning of vaginal tissues can make them more susceptible to irritation and inflammation, resulting in itching and a burning sensation. Vaginal atrophy can cause dyspareunia due to reduced lubrication and thinning of the vaginal wall, and can also contribute to urinary issues such as increased frequency, increased urgency, and recurrent urinary tract infections. The most effective treatment for VVA is often estrogen replacement therapy. This can be administered topically in the form of creams, rings, or tablets to directly address the local tissues without significant systemic absorption. Regular sexual activity, including stimulation and intercourse, can help maintain vaginal health by promoting blood flow and preventing the atrophy of tissues. Pelvic floor exercises, such as Kegel exercises, may help improve pelvic floor tone and alleviate some symptoms. Therefore, local estrogens in the form of creams, pessaries, tablets, and the estradiol-releasing ring represent the first-line therapy [16]. To offer clinicians and women an improved non-drug option for managing symptoms connected to VVA, the microablative fractional CO_2_ laser has emerged and has been utilized in recent years [17,18].

Primarily utilized in skin rejuvenation treatments, the fractional microablative CO_2_ works by creating micro-columns of ablation that remove small skin areas. This action triggers the body’s natural healing response, leading to an increase in collagen production. As a result, there is a noticeable improvement in the skin’s texture, tone, and elasticity, making it highly effective against wrinkles, fine lines, and sun damage [17,18,19,20,21,22,23]. In the realm of gynecology, the fractional microablative CO_2_ laser has sparked interest due to its potential in treating various vaginal health conditions. Notably, it has been employed in addressing issues such as the genitourinary syndrome of menopause (GSM), vaginal atrophy, and urinary incontinence. The treatment involves a specialized CO_2_ laser device, which emits controlled and fractional laser energy to the target tissues, with effects similar to those observed in the vaginal canal [24]. This fractional method ensures that the surrounding tissues remain intact, which promotes faster healing. The laser’s impact extends to tissue remodeling and scar tissue regeneration. Its application in treating atrophic skin has demonstrated its capability to regenerate scar tissue. This treatment causes significant histological changes in connective elements. The laser’s mechanism includes a microablative process that initiates tissue remodeling [25,26], akin to the regeneration of scar tissue. Tissue remodeling can be triggered by various stimuli, such as injuries, inflammation, or changes in mechanical forces. For instance, in wound healing, the initial response involves blood clot formation and the release of signaling molecules such as growth factors and cytokines. In response to injuries or stress, an inflammatory phase begins. Immune cells, including neutrophils and macrophages, infiltrate the affected tissue, aiding in the removal of debris, pathogens, and damaged cells, thus creating an environment conducive to tissue repair. The microablative action, as shown in skin treatments [27], locally increases several cytokines, particularly transforming growth factor-alfa (TGF-α), basic fibroblast growth factor (bFGF), epidermal growth factor (EGF), platelet-derived growth factor (PDGF), and vascular endothelial growth factor (VEGF). These factors activate fibroblasts to produce new collagen, other extracellular matrix components (proteoglycans, glycosaminoglycans, and other molecules), and new blood vessels, specifically impacting epithelial tissue [28]. However, the safety and efficacy of this procedure can depend on individual health factors and the specific condition being addressed. There are also contraindications for fractional microablative CO_2_ laser therapy, including pregnancy, active infections in the treatment area, a history of keloid formation, immunosuppression, previous radiotherapy, clotting disorders, uncontrolled diabetes, photosensitivity, active Herpes Simplex Virus infection, and a history of hypertrophic scarring. Individuals considering fractional microablative CO_2_ laser therapy must undergo a comprehensive evaluation by a qualified healthcare provider. The provider will evaluate the patient’s medical history and current health condition, along with any possible contraindications, to ascertain whether the procedure is appropriate and to ensure patient safety. Based on the aforementioned factors, this study aimed to evaluate the impact of fractional CO_2_ laser treatment of pelvic symptoms in women who have experienced previous perineal trauma due to vaginal childbirth.

## 2. Materials and Methods

The research conducted was a retrospective, monocentric analysis involving all women who underwent evaluation for pelvic discomfort or signs of VVA at the Institute for Social Security of the Republic of San Marino from 2013 to 2018 and who had previously given birth vaginally at the same hospital. These patients received fractional CO_2_ laser treatment (Monalisa Touch, DEKA, Florence, Italy) due to ongoing symptoms or signs that persisted despite undergoing estrogen treatment, contraindications to estrogen therapy, or a personal decision to decline such treatment. Each session utilized a power setting of 30 watts, delivering energy at a density of 5 MJ/cm^2^. The laser operated in a pulsed mode, with a pulse duration of 200 µs. Patients underwent a total of three treatment sessions, each spaced four weeks apart to allow for adequate tissue healing and response evaluation. The laser application involved a systematic approach, covering the entire affected perineal area with particular attention to areas with scar tissue from previous perineal trauma. The laser’s microablative action was aimed at stimulating collagen remodeling and promoting tissue healing and regeneration. They had undergone the following tests during the preparation for laser treatment: complete blood count and coagulation, blood glucose, serum beta-HCG, urinalysis and eventually urine culture, and vaginal and cervical cultures. Only patients meeting the following criteria were included: negative serum beta-HCG test; absence of a history of prior perineal surgery, keloids, immunosuppression, radiation therapy, coagulopathy, diabetes, or photosensitivity; negative urinalysis or eventually urine culture; negative vaginal and cervical cultures.

Twenty-seven patients fulfilled the inclusion criteria and were segregated into two study cohorts: (1) individuals who underwent an episiotomy during labor (*n* = 11); and (2) those who encountered spontaneous lacerations during vaginal delivery (*n* = 16).

Symptom intensity was evaluated utilizing the Visual Analogue Scale (VAS), a linear scale denoting extreme limits from “no symptom at all” to “symptom as severe as possible” [29].

Information was gathered from an electronic repository encompassing demographic, obstetric, and overall details about the women and their deliveries. Access to this database was feasible because the Institute for Social Security is the sole medical institution catering to the country’s population, handling all local deliveries within its labor and delivery suites. The Department of Obstetrics and Gynecology maintains a computerized database encompassing records of all deliveries. Trained secretaries transcribe information from patient medical records and code it following ICD-9 diagnoses.

The Ethics Committee of San Marino waived the requirement of ethical approval because the study used previously archived data. Records/information were anonymized and de-identified before analysis. Informed consent was obtained from all subjects involved in the study.

The information was processed through SPSS 21.0 (SPSS, Chicago, IL, USA). The initial assessment involved descriptive statistics. Categorical variables were articulated in counts and percentages, and their statistical assessment relied on chi-square or the Fisher exact test as suitable. Symptom scores across different groups were compared using the Mann–Whitney test. To gauge the personalized effectiveness of treatment, each group was individually compared, with each woman acting as her control, utilizing the Wilcoxon signed-rank test. Any analyses yielding a two-sided *p*-value of <0.05 were deemed statistically significant.

## 3. Results

The demographic traits exhibited likeness between the study groups. However, women who underwent an episiotomy showed a notably higher incidence of prior cesarean section compared to those who encountered a spontaneous perineal laceration during vaginal delivery (27.3% vs. 0.0%, *p* = 0.02) (Table 1).

Assessing the impact of treatment of symptoms reported by women who had undergone episiotomies during previous vaginal deliveries revealed consistent and substantial enhancements. Comparisons between each treatment session and the subsequent post-treatment control consistently showcased significant improvements in dyspareunia intensity (8 vs. 6 after the first treatment, *p* = 0.01; 6 vs. 4 after the second treatment, *p* = 0.01; 4 vs. 1 after the third treatment, *p* = 0.01) and pain at the introitus (7.5 vs. 6.5 after the first treatment, *p* = 0.03; 6.5 vs. 3 after the second treatment, *p* = 0.01; 3 vs. 1 after the third treatment, *p* = 0.01). Dryness exhibited improvement post-first treatment (5.5 vs. 4, *p* = 0.04) and post-second treatment (4 vs. 2, *p* = 0.04), while the intensity remained relatively unchanged after the third treatment in women requiring it. This became significant when taking into consideration the comparison between intensity before the first treatment and after the last treatment (5.5 vs. 2, *p* = 0.02) (Table 2).

Women who encountered spontaneous perineal lacerations during vaginal delivery demonstrated noteworthy enhancements in dyspareunia following all treatment sessions (8 vs. 7 after the first treatment, *p* = 0.01; 8 vs. 4 after the second treatment, *p* = 0.02; 3 vs. 1 after the third treatment, *p* = 0.03). However, only the first (8 vs. 7, *p* = 0.03) and the second treatments (8 vs. 3, *p* = 0.03) exhibited significant effects on pain at the introitus. The favorable impact on dryness was observed solely after the initial treatment (6.5 vs. 3.5, *p* = 0.02). Of interest, women undergoing episiotomy displayed significant improvements in dyspareunia, pain at the introitus, and dryness when comparing the intensity of these symptoms before the first treatment and after the final treatment (Table 3).

The effect of laser treatment did not show significant differences when women undergoing episiotomy were compared to those experiencing spontaneous perineal lacerations (Table 4).

## 4. Discussion

The present investigation indicates that employing the CO_2_ laser resulted in improvements in dyspareunia, pain at the vaginal introitus, and dryness linked to VVA. These improvements were observed in both women who underwent episiotomies and those who experienced spontaneous perineal lacerations during delivery. Interestingly, the impact of CO_2_ laser treatment did not exhibit variation based on the type of lacerations—whether women underwent episiotomies or experienced spontaneous perineal lacerations.

The employment of laser treatment in women with symptoms of VVA has different benefits. CO_2_ laser therapy offers a non-hormonal option for women who cannot or prefer not to use hormonal treatments for vaginal atrophy. Laser therapy emerged as an alternative devoid of hormones to address GSM treatment. This method operates by triggering the body’s innate repair, growth, and healing mechanisms, thereby expediting tissue regeneration. The microablative fractional CO_2_ laser and the Erbium: YAG (Er: YAG) laser are two main types used to treat GSM. The standard treatment with these lasers typically involves three sessions, spaced 4–6 weeks apart. The CO_2_ laser, with a 10,600 nm wavelength, varies in tissue penetration depth and ablation due to tissue water absorption. In contrast, the Er: YAG laser operates at 2940 nm, offering more precise ablation and deeper thermal effects. These treatments, reported to improve vaginal dryness, itching, and pain, are considered safe when conducted by skilled healthcare professionals. In addition, some studies suggest that CO_2_ laser therapy may help improve urinary symptoms associated with GSM, such as urinary urgency and frequency. The principle of laser treatment is based on hitting the matrix of subcutaneous tissue, creating small holes in the subcutaneous tissue matrix. This method aims to enhance blood supply and improve the morphology of the vaginal epithelium. Consequently, this process aids in alleviating symptoms of vaginal atrophy during the repair phase. Based on this reasoning, Pagano et al.’s study involved 33 post-menopausal women with VVA symptoms who underwent laser treatment. Short-term findings indicated that three weekly treatment sessions effectively ameliorated subjective symptoms such as dryness, burning, and dyspareunia, alongside visible clinical improvements. Notably, 90% of patients expressed satisfaction with the procedure and reported significant enhancements in their quality of life. Importantly, no adverse events were documented during the study [30].

Various literature reviews have proposed that using CO_2_ laser technologies intravaginally may mitigate symptoms associated with VVA [28]. In 2017, a meta-analysis scrutinized available evidence on the efficacy and safety of intravaginal laser therapy, involving 14 studies encompassing 542 eligible women. Notably, none of these studies constituted randomized controlled trials. Among them, 12 studies were retrospective, comparing outcomes before and after treatment, while only two studies prospectively compared results with an alternative therapy (local estriol cream). All studies examined the impact of three weekly laser sessions. Results from the meta-analysis revealed a substantial improvement in primary outcomes across all studies within one month. Moreover, there was a reduction in urinary incontinence rates observed at the 1-month follow-up, maintained for up to 6 months. However, the assessment of evidence quality for dryness, dyspareunia, and incontinence was rated as “low”, and for itching, burning, and dysuria, even “very low”. In a subgroup analysis focusing on CO_2_ laser treatment, the combined mean difference for dryness and dyspareunia was −5.5 (95% CI: −6.6 to −4.4; *p* < 0.00001, I2: 0%; *n* = 255) and −5.5 (95% CI: −6.6 to −4.4; *p* < 0.00001; I2: 0%; *n* = 229), respectively. The difference in VAS 0–10 scores for dryness and dyspareunia remained significant even after a 3-month follow-up. Notably, only two out of the 542 patients included in the analysis discontinued treatment after the first session due to discomfort, primarily a burning sensation [31]. Additionally, several secondary outcomes were taken into consideration and evaluated as urinary incontinence (appraised through micturition diaries), dyspareunia and dryness (gauged using the VAS), itching, burning, and dysuria (evaluated using the VAS 0–10), frequency and urgency of urination, and urinary tract infections (monitored using urine culture). Furthermore, secondary outcomes encompassed the assessment of adverse events and participant dropouts due to adverse events. Observations of histopathological alterations in the vaginal mucosa persisted for up to 12 months of follow-up (two studies, *n* = 11). Specifically, there was noted augmentation in the thickness of the vaginal epithelium, coupled with enhancements in vascularization and the penetration of angiogenesis into new papillary formations. Moreover, an increase in the number of fibroblasts and the synthesis of extracellular matrix fibrillar components was evident. Furthermore, a histopathological investigation assessing the impact of CO_2_ laser treatment revealed heightened levels of glycogen stored within large epithelial cells, along with an increase in the shedding of superficial epithelial cells. This study emphasized the distinct impact of fractional CO_2_ laser application on the vaginal mucosa, specifically noting microscopic and ultrastructural alterations that correlate closely with the wavelength, pulse profile, and pulse width utilized. The 10,600 nm wavelength selectively targets mucosal tissues, resulting in minimal nonspecific thermal damage, especially when combined with a specialized pulse profile designed to treat the vaginal mucosa. This profile involves an initial high peak power followed by a longer low-power phase, allowing the laser to primarily ablate the surface epithelial layer while releasing sub-ablative thermal energy into the underlying connective tissue without causing uncontrolled thermal harm. These outcomes are achieved using a specially designed opto-mechanical scanner that distributes the laser beam spots in a specific pattern. The findings strongly supported the idea that this laser application prompts regeneration of collagen, ground substances in connective tissue, and glycogen and acidic mucins in the epithelium. This rejuvenation process aids in restoring and rebalancing the vaginal mucosa affected by estrogen-deficiency-induced atrophy, leading to a substantial enhancement in clinical symptoms. Ultimately, the results proposed promising avenues for utilizing electromagnetic radiation to reinstate both the structural and physiological integrity of atrophic vaginal mucosa [17].

In the context of alternative or complementary treatments to laser therapy for pelvic symptoms, Platelet-Rich Plasma (PRP) therapy emerges as a noteworthy option. PRP therapy, which concentrates growth factors from the patient’s blood, has shown promise in enhancing tissue repair and regeneration. These growth factors, including PDGF, transforming growth factor-beta (TGF-β), and VEGF, are known to stimulate cellular proliferation and angiogenesis [32]. This biological stimulation could complement the microablative effects of laser therapy, potentially leading to more effective healing and tissue regeneration [33]. The synergy between PRP’s regenerative capabilities and the precision of laser therapy in remodeling and rejuvenating tissue could offer a new paradigm in the treatment of pelvic floor disorders. This combination therapy might be especially advantageous for patients with severe symptoms or those who have exhibited limited response to conventional treatments [33]. However, the application of PRP in conjunction with laser therapy is still in its nascent stages, and there are significant gaps in the understanding of the optimal protocols and long-term effects. Rigorous clinical trials are necessary to establish the efficacy, safety, and best practice guidelines for this combined therapeutic approach.

Several constraints exist within this study, notably its retrospective design and the relatively small sample size. To overcome these limitations, future investigations might benefit from larger cohorts and the implementation of longitudinal studies that track women from pregnancy through the postpartum period. 

However, the fact that the procedure was performed in a single center, representing the only hospital in the area, reduces the risk of missing patients and data and is a point of strength of this study. Prospective, multicenter studies are needed to validate these results.

In conclusion, fractional CO_2_ laser has a beneficial effect on cutaneous, urinary, and sexual pelvic symptoms in women with previous perineal trauma associated with vaginal delivery, independently from the nature, spontaneity, or iatrogenesis of the perineal laceration.

## Figures and Tables

**Table 1:** Demographic characteristics.

Characteristics	Women Undergoing Episiotomy (*n* = 11)	Women Undergoing Spontaneous Laceration (*n* = 16)	*p*-Value
Age			
<21	0 (0.0%)	0 (0.0%)	
21–35	7 (63.6%)	9 (56.3%)	0.50
>35	4 (36.4%)	7 (43.7%)	
Parity			
0	1 (9.1%)	0 (0.0%)	
1	6 (54.5%)	8 (50.0%)	0.57
2–4	4 (36.4%)	8 (50.0%)	
History of cesarean section	3 (27.3%)	0 (0.0%)	0.02
Dyspareunia	9 (81.8%)	13 (81.3%)	0.68
Introitus	10 (90.9%)	9 (56.3%)	0.06
Dryness	8 (72.7%)	8 (50.0%)	0.22
Pruritus	2 (18.2%)	5 (31.3%)	0.38
Burning	4 (36.4%)	4 (25.0%)	0.14
Heat	0 (0.0%)	2 (12.5%)	0.34
Urinary stress incontinence	2 (18.2%)	2 (12.5%)	0.54

Data are presented as number (percentage), mean ± standard deviation.

**Table 2:** Evaluation of treatment efficacy in women undergoing episiotomy.

Symptoms	Before the Treatment	After the Treatment	*p*-Value
Dyspareunia	8 (7–10)	6 (4.5–8)	0.01
Introitus	7.5 (5.25–9.25)	6.5 (3.5–7.25)	0.03
Dryness	5.5 (3.5–8.75)	4 (2–7.75)	0.04
Pruritus	5.5 (3–5.5)	1 (0–1)	0.18
Burning	4.5 (1.75–8.75)	1.5 (0.25–8)	0.18
Heat	-	-	n/a
Dyspareunia	6 (4.5–8)	4 (2.5–5.5)	<0.01
Introitus	6.5 (3.5–7.25)	3 (1.5–5–25)	<0.01
Dryness	4 (2.5–7.75)	2 (0.6.5)	0.04
Pruritus	1 (0–1)	0 (0–0)	0.32
Burning	1.5 (0.25–8)	0 (0–5.25)	0.11
Heat	-	-	n/a
Dyspareunia	4 (2.5–5.5)	1 (0–3.5)	0.01
Introitus	3 (1.5–5.25)	1 (0–2.5)	0.01
Dryness	2 (0–6.5)	2 (0–4.5)	0.18
Pruritus	0 (0–0)	0 (0–0)	1.00
Burning	0 (0–5.25)	0 (0–0)	0.32
Heat	-	-	n/s
Dyspareunia	8 (7–10)	1 (0–3.5)	<0.01
Introitus	7.5 (5.25–9.25)	1 (0–2.5)	<0.01
Dryness	5.5 (3.5–8.75)	2 (0–4.5)	0.02
Pruritus	5.5 (3–5.5)	0 (0–0)	0.18
Burning	4.5 (1.75–8.75)	0 (0–0)	0.07
Heat	-	-	n/a

n/a: not applicable. Data are presented as median and interquartile range.

**Table 3:** Evaluation of treatment efficacy in women undergoing spontaneous perineal lacerations.

Symptoms	Before the Treatment	After the Treatment	*p*-Value
Dyspareunia	8 (6–10)	7 (1.5–8)	<0.01
Introitus	8 (7.5–10)	7 (3–8.5)	0.03
Dryness	6.5 (5–7.75)	3.5 (2–6.25)	0.02
Pruritus	5 (2.5–7)	5 (1.5–6)	0.16
Burning	8 (2.75–9.5)	0.5 (0–4)	0.07
Heat	4.5 (1–4.5)	2.5 (0–2.5)	0.18
Dyspareunia	8 (7–9)	4 (2–7)	0.02
Introitus	8 (5.75–9.25)	3 (1.5–6.25)	0.03
Dryness	5 (2.25–7)	2 (0.5–5.75)	0.18
Pruritus	3.5 (1.25–5)	1.5 (0–4.5)	0.10
Burning	0.5 (0–0.5)	0 (0–0)	0.32
Heat	0 (0–0)	0 (0–0)	1.00
Dyspareunia	3 (2.6.25)	1 (0–4.25)	0.03
Introitus	2 (1.5–5)	0 (0–3)	0.06
Dryness	2 (0–2)	1 (0–1)	0.32
Pruritus	3 (0–3)	3 (0–3)	1.00
Burning	0 (0–0)	0 (0–0)	1.00
Heat	0 (0–0)	0 (0–0)	1.00
Dyspareunia	8 (6–10)	1 (0–4.5)	<0.01
Introitus	8 (7.5–10)	1 (0–5.5)	<0.1
Dryness	6.5 (5–7.75)	2 (1–4)	0.01
Pruritus	5 (2.5–7)	3 (0–6)	0.06
Burning	8 (2.75–9.5)	0 (0–3.75)	0.07
Heat	4.5 (1–4.5)	2.5 (0–2.5)	0.18

Data are presented as median and interquartile range.

**Table 4:** Symptoms assessment before and after treatment.

Characteristics	Women Undergoing Episiotomy (*n* = 11)	Women Undergoing Spontaneous Laceration (*n* = 16)	*p*-Value
One treatment	11 (100.0%)	16 (100.0%)	0.49
Two treatments	11 (100.0%)	10 (62.5%)	
Three treatments	11 (100.0%)	8 (50.0%)	
Symptoms before first treatment			
Dyspareunia	9 (7–10)	8 (6–10)	0.95
Introitus	7.5 (5.25–9.25)	8 (7.5–10)	0.28
Dryness	5.5 (3.5–8.75)	6.5 (5–7.75)	0.80
Pruritus	5.5 (3–5.5)	5 (2.5–7)	1.0
Burning	4.5 (1.75–8.75)	8 (2.75–9.5)	0.68
Heat	-	4.5 (1–4.5)	n/a
Urinary stress Incontinence	3 (3–3)	3 (3–3)	1.0
Symptoms at first control			
Dyspareunia	6 (4.5–8)	7 (1.5–8)	0.60
Introitus	6.5 (3.5–7.25)	7 (3–8.5)	0.60
Dryness	4 (2–7.75)	3.5 (2–6.25)	0.65
Pruritus	1 (0–1)	5 (1.5–6)	0.19
Burning	1.5 (0.25–8)	0.5 (0–4)	0.49
Heat	-	2.5 (0–2.5)	n/a
Symptoms before second treatment			
Dyspareunia	6 (4.5–8)	8 (7–9)	0.25
Introitus	6.5 (3.5–7.25)	8 (5.75–9.25)	0.18
Dryness	4 (2–7.75)	5 (2.25–7)	0.93
Pruritus	1 (0–1)	3.5 (1.25–5)	0.27
Burning	1.5 (0.25–8)	0.5 (0–0–5)	0.53
Heat	-	0 (0–0)	n/a
Symptoms at second control			
Dyspareunia	4 (2.5–5.5)	4 (2–7)	0.84
Introitus	3 (1.5–5.25)	3 (1.5–6.25)	0.87
Dryness	2 (0–6.5)	2 (0.5–5.75)	0.93
Pruritus	0 (0–0)	1.5 (0–4.5)	0.53
Burning	0 (0–5.25)	0 (0–0)	1.0
Heat	0 (0–0)	-	n/a
Symptoms before third treatment			
Dyspareunia	4 (2.5–5.5)	3 (2–6.25)	0.86
Introitus	3 (1.5–5.25)	2 (1–5.5)	0.86
Dryness	2 (0–6.5)	2 (0–2)	0.63
Pruritus	0 (0–0)	3 (0–3)	0.40
Burning	0 (0–5.25)	0 (0–0)	1.0
Heat	-	(0–0)	n/a
Symptoms at third control			
Dyspareunia	1 (0–3.5)	1 (0–4.25)	1.0
Introitus	1 (0–2.5)	0 (0–3)	0.51
Dryness	2 (0–4.5)	1 (0–1)	0.49
Pruritus	0 (0–0)	3 (0–3)	0.40
Burning	0 (0–0)	0 (0–0)	1.00
Heat	-	0 (0–0)	n/a

n/a: not applicable. Data are presented as number (percentage), median, and interquartile range.

## Data Availability

The data presented in this study are available on request from the corresponding author.
